# Direct electric field imaging of graphene defects

**DOI:** 10.1038/s41467-018-06387-8

**Published:** 2018-09-24

**Authors:** Ryo Ishikawa, Scott D. Findlay, Takehito Seki, Gabriel Sánchez-Santolino, Yuji Kohno, Yuichi Ikuhara, Naoya Shibata

**Affiliations:** 1 Institute of Engineering Innovation, University of Tokyo, Bunkyo, Tokyo 113-8656 Japan; 2 School of Physics and Astronomy, Monash University, Victoria, 3800 Australia; 3 Electron Optics Division, JEOL Ltd., Akishima, Tokyo 196-855 Japan; 4 Nanostructures Research Laboratory, Japan Fine Ceramics Center, Nagoya, Aichi 456-8587 Japan

## Abstract

Material properties are sensitive to atomistic structure defects such as vacancies or impurities, and it is therefore important to determine not only the local atomic configuration but also their chemical bonding state. Annular dark-field scanning transmission electron microscopy (STEM) combined with electron energy-loss spectroscopy has been utilized to investigate the local electronic structures of such defects down to the level of single atoms. However, it is still challenging to two-dimensionally map the local bonding states, because the electronic fine-structure signal from a single atom is extremely weak. Here, we show that atomic-resolution differential phase-contrast STEM imaging can directly visualize the anisotropy of single Si atomic electric fields in monolayer graphene. We also visualize the atomic electric fields of Stone–Wales defects and nanopores in graphene. Our results open the way to directly examine the local chemistry of the defective structures in materials at atomistic dimensions.

## Abstract (web summary)

Imaging chemical bonding states in defective graphene is important to determine its functional properties. Here, the authors report triangular and rectangular atomic electric fields in monolayer graphene induced by silicon as imaged by differential phase contrast STEM.

## Introduction

Graphene is an atomically thin two-dimensional (2D) material, exhibiting a vast range of prominent physical and chemical properties such as mechanical, electric, magnetic, and catalytic properties^1–4^. Pure graphene consists of *sp*^2^ hybridized carbon atoms arranged in a honeycomb lattice, but synthesis inevitably introduces a wide variety of atomistic structure defects including impurity dopants, vacancies, non-hexagonal polygons, edges, and nanopores^5^. Although these structural defects often adversely affect the mechanical or electronic transport properties, graphene edges and nanopores have potential for applications in chemical separation, water-splitting, DNA sequencing, and catalysis^3^.

Low voltage (< 80 kV) annular dark-field scanning transmission electron microscopy (ADF-STEM) imaging has greatly contributed to the direct identification of individual elements and their locations inside defects in 2D materials^6^. Moreover, the electronic structure can be probed at the atomic level by incorporating electron energy-loss spectroscopy (EELS) in STEM^7–9^. The energy-loss near-edge structure (ELNES) is correlated to the local chemical bonding states. However, ELNES signals are extremely weak and it is still challenging to obtain atomic-resolution 2D ELNES maps, especially at defective regions, because the specimen is unstable under the beam irradiation and mechanical instability of the microscope hinders using very long acquisition times. Apart from spectroscopic analysis, differential phase-contrast (DPC) STEM imaging has recently been shown to be capable of visualizing the electric field in atoms^10–12^. According to the literature by Linus Pauling^13^, a chemical bond can be interpreted as the forces acting between atoms, and therefore the direct imaging of electric fields (force fields for electric charges) with DPC–STEM may experimentally probe the local chemical bonding states in two dimensions.

Here, we show atomic-resolution electric field imaging at dopants and topological defects in graphene by using DPC–STEM recorded with a second-generation segmented annular all-field detector (SAAF, 16 segment elements)^10,14^, operating the microscope at 80 kV (JEOL ARM300CF installed at the University of Tokyo)^15^. We note that the present electric field imaging requires a relatively higher spatial resolution than the ADF-STEM image, because the chemical bonding information is present in the signal shape in the space between the atom positions. Therefore, we operated the microscope at 80 kV, which is smaller than the knock-on threshold of 86 kV for carbon atoms. By properly choosing the electron microscope operating condition and the detector geometry, we elucidate the anisotropy of single Si atomic electric fields located in the coordination of threefold and fourfold symmetry in graphene. Furthermore, we also identify the local enhancement or suppression of electric fields of topological defects such as Stone–Wales defects and nanopores in graphene. This result opens a new capability to investigate the local chemistry of atomistic defects in materials and might shed light on our understanding of material properties at the level of single atoms.

## Results

### Atomic-resolution DPC–STEM imaging of monolayer graphene

When an atomically focused electron probe is traveling through the atoms in materials, a fraction of the incident electrons is incoherently scattered into high angles. This imaging mode is known as ADF-STEM, which can be used to directly determine the location of the atoms and is also sensitive to the chemical type (Z-contrast, where Z is the atomic number)^16^. However, it is the coherently scattered electrons in the bright-field region that are expected to contain rich information on the local chemical bonding states. In DPC–STEM, the segmented detectors are set in the bright-field region^10,12^ and thus permit direct visualization of the local electric fields at atomic dimensions, where the electric field is detected as the variation of the center of mass (CoM) in the diffraction pattern for each probe position^11,17^. A major issue for reliable atomic electric field imaging in DPC–STEM is dynamical diffraction: when a specimen has heavy elements or several-nanometer thickness^17,18^, the electric field estimated from the measured CoM in the diffraction plane may deviate significantly from the actual electric field. As graphene is composed of light carbon atoms with single-atom thickness, dynamic diffraction is negligible, and so graphene is an ideal specimen for quantitative electric field imaging in DPC–STEM. However, this requires a trade-off between the experimental signal and specimen damage: a higher electron dose is required to reliably detect the very tiny CoM signal from the weakly scattering carbon atoms in graphene. Even at accelerating voltages lower than the knock-on threshold (< 86 kV), dopants or topological defects in graphene seldom survive high electron dose or prolonged beam irradiation^19^. To overcome the specimen damage while maintaining a good signal-to-noise ratio (SNR), we performed sequential fast-scanning DPC–STEM imaging with the scintillator-type segmented detector, which suppresses both the electron dose rate limited damage and severe specimen drift^12,20^.

**Fig. 1:**
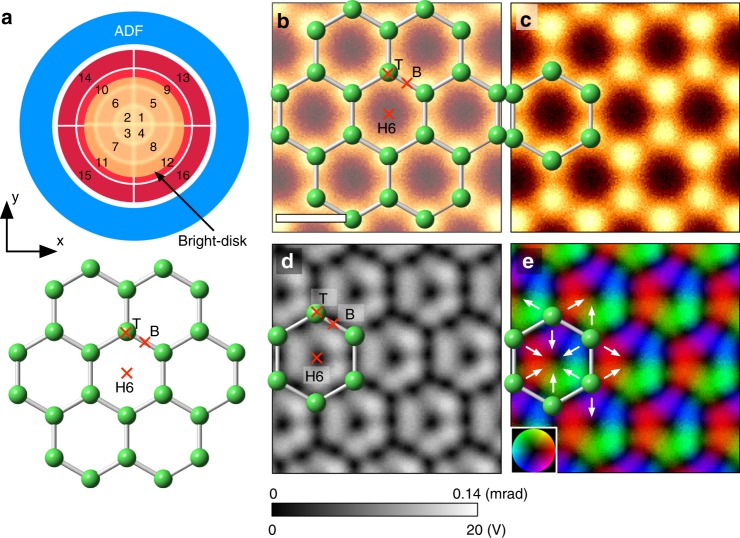
Projected atomic electric field maps of monolayer graphene. **a** Schematic view of the orientation relation between the segmented/ADF detectors and the crystal of monolayer graphene. The yellow disk indicates the bright-field region. **b**, **c** Atomic-resolution ADF-STEM image, **d** projected electric field strength map, and **e** projected electric field vector map. The contrast range in (**d**) is 0–20 V or 0–0.14 mrad. The scale bar in (**b**) is 2 Å

Figure 1a shows the experimental orientation relationship between the 16 segmented detectors and the monolayer graphene sample, with the bright-field disk given by the yellow disk in the middle of the third ring. Figure 1b, c shows an ADF-STEM image of monolayer graphene and the corresponding atomic structure model, where the bright contrast corresponds to the positions of the carbon atoms. Since the experimental projected electric field is integrated along the [0001] direction, we use the unit of volt (V) for the projected electric field (it is also possible to use V m^−1^ by dividing the specimen thickness). Figure 1d shows the projected electric field strength map constructed from the 16 segmented detector images, which is blurred by the effective source size of the electron probe. Since the electric field of an isolated single atom points radially outward from the nucleus, the projected electric field strength map for an isolated atom would show a donut-shape contrast, with the projected electric field minimized at the center of the atom (nuclear site)^12^. For an ionic crystal, the electric field can be approximated to be the superposition of the atomic electric fields of the isolated atoms, and the electric-field sharp minima appear only at the nuclear positions. However, the observed electric field strength map of graphene has a rather complicated intensity distribution: in addition to the nuclear positions (T-site: top site), several electric field minima appear in-between atoms as dark dot contrast. The minimum electric fields appear at the high symmetry points of the carbon hexagon: the hexagon center (H6-site: hollow site) and the midpoints of the hexagon ridges (B-site: bridge site). Owing to the strong covalent bonding between carbon atoms, the interatomic distance is as small as 142 pm, with the consequence that the atomic electric fields of adjacent carbon atoms partially overlap, leading to the emergence of the electric field minima at the high symmetry points. For example, at the B-site, the electric fields of the two neighboring carbon atoms have exactly the same strength but in the opposite direction, and consequently the net projected electric field cancels out (ideally to zero, but the extent to which this is recorded in an experiment is limited by shot noise and residual aberrations). Similarly, the net projected electric field at the H6-site is also a minimum as a result of the superposition of the electric fields of the surrounding six carbon atoms. Figure 1e shows the projected electric field vector map, in which the color contrast indicates the relative direction and the strength of the electric field. The white arrows indicate the direction of the projected electric field. Since the atomic electric field along the covalent bonding direction is considerably suppressed, the atomic electric field is anisotropic and relatively stronger along the directions shown by the white arrows than that along chemical bonds.

To demonstrate the advantages of atomic electric field imaging, we consider the problem of finding the stable adsorption sites for adatoms on graphene. Since the forces of an adatom on graphene are minimized at a stable absorption site, a 2D force map may be used to find minimum force positions for the adatom. Usually, the single atoms of metallic elements lose their outer electrons easily, while the atoms of nonmetallic elements obtain additional electrons^13^, acting as cations and anions, respectively. We then approximate these single anions or cations to be infinitesimal positive or negative charges, and therefore the observed graphene electric field can be considered as a force field for the single adatom at each position (Hellman–Feynman theorem^21^). On the basis of the observed graphene electric field image, the stable adsorption sites for the adatom are expected to be the positions of the minimum electric field or dark contrast in Fig. 1d, i.e., T-, B-, or H6-sites. On close inspection of the local electric field direction, the electric fields at T- and B-sites point outward (slightly inward along the bonding direction at B-sites), while the electric field at H6-site points radially inward. A single cation adatom on the graphene surface feels the electrostatic force along the white arrow directions in Fig. 1e and would be stabilized at H6-site. Conversely, since an anion has negative charge and the electrostatic force for an anion is thus in the opposite direction to the white arrows in Fig. 1e, the anion would be stable at B- or T-sites. However, since the T-site has a strong electric field parallel to the incident electron beam direction (the net electric field in projection becomes minimum), the anions might be stable at B-sites to minimize the force. In the previous literature, density functional theory (DFT) calculations suggest that almost all the metal (cation) adatoms are stable at the H6-site, except for some noble metals (Cu, Pd, Ag, Pt, and Au), while most of the nonmetallic (anion) adatoms are stable at the B-site^22^, which strongly supports our prediction. Graphene is a relatively simple system and DFT calculation can precisely predict the stable adatom site. However, DFT calculations may be difficult to perform in a large, defective, or complex materials system, and therefore the present view of experimental force field imaging could be useful for the further prediction of a local chemical reaction (although the prediction may not be perfect: the accuracy for the case of graphene adatoms is ∼90%). From these considerations, DPC–STEM images can be considered as a force field for electric charges and hence this imaging mode may be said to enable visualization of the anisotropic force field between atoms, i.e., chemical bonding.

### Anisotropic single Si atomic electric fields in graphene

**Fig. 2:**
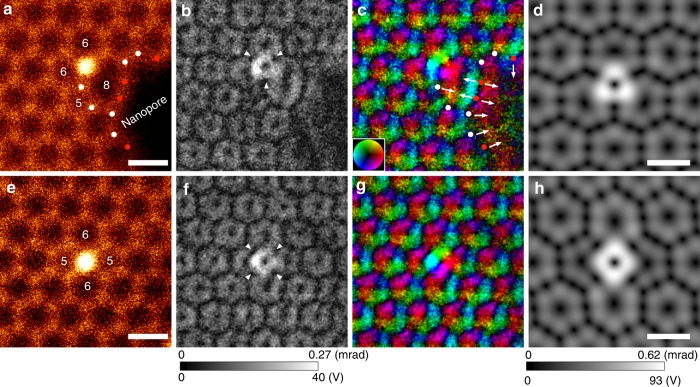
Experimental and simulated projected atomic electric field maps of single Si dopants in graphene. **a**, **e** ADF-STEM images, **b**, **f** electric field strength maps, **c**, **g** electric field vector maps, **d**, **h** calculated electric field strength maps from simulated STEM images for the 16 segmented detectors, in the coordination of Si–C_3_ and Si–C_4_, respectively. The contrast ranges in (**b**), (**f**) and (**d**), (**h**) are 0–40 V and 0–93 V, respectively. The numbers in (**a**), (**e**) indicate the number of members in the ring. The scale bars in (**a**), (**d**), (**e**), (**h**) are 2 Å.

To observe anisotropy in atomic electric fields, we selected single Si dopants in graphene, where there were two types of stable Si point defect configurations: threefold- (Si–C_3_, substituting for a single carbon atom) and fourfold- (Si–C_4_, substituting for two carbon atoms) coordinated Si dopants. As per previous EELS experiments^8,9^, the single Si dopant in threefold or fourfold coordination forms a different chemical bonding state of *sp*^3^ or *sp*^2^*d*, respectively, suggesting that the Si atomic electric field also depends on these defect configurations. Figure 2, for threefold- and fourfold-coordinated Si defects in graphene, respectively, show the simultaneously recorded ADF-STEM images, and the projected electric field strengths and vector maps. We note that Si defects or carbon vacancies in graphene are usually unstable even under the low-voltage beam irradiation at 60 kV with higher beam current or longer dwell time per pixel^23,24^, and it is therefore necessary to quickly acquire images before defect-diffusion or defect-assisted graphene etching occurs. A series of fast-scanning DPC–STEM images (20 µs/pixel) were recorded (512×512 pixels, eight frames, total acquisition time 43 s) and then averaged after image alignment via cross-correlation^20^. Since Si is much heavier than C, the strong electric fields of single Si atoms can be recognized as a donut-like ring of bright contrast in Fig. 2b, f. However, the Si atomic electric field strength is not circularly symmetric but rather has threefold (triangular) and fourfold (square) point symmetry for Si–C_3_ and Si–C_4_ configurations, respectively. The atomic electric fields of Si and C have an opposite sign along the covalent bonding direction and therefore the Si atomic electric field is suppressed along the C bonding directions (white arrowheads in Fig. 2b, f, see also Supplementary Figure 1). Consequently, the Si atomic electric fields become upper-triangular in shape in the threefold configuration and square in shape in the fourfold configuration.

To confirm the validity of the observed Si atomic electric fields, we performed image simulations at the experimental optical condition^17^, using the structure models of Si–C_3_ and Si–C_4_ configurations relaxed by DFT calculations. Figure 2d, h shows the electric field strength maps calculated from the simulated 16 segmented detector images, where these maps were convolved with the experimental effective source size. The simulated electric field strength maps well reproduce the observed anisotropy of Si atomic electric fields, which are the consequence of the formation of the local chemical bonding. We note that since the electric field imaging uses a bright-field disk region, the resultant intensity is quite sensitive to the residual aberrations, leading to the imperfection of threefold and fourfold symmetry and a reduction of the measured strength of the Si atomic electric fields. Therefore, the discrepancy of the projected electric field strength between experiment and simulation could originate from residual aberrations.

The atomic electric field strength at defects may be qualitatively described by the simple parameters of the interatomic distance, coordination number, and neighbor atom type. When an atom is located in an environment with a shorter interatomic distance or a larger coordination number than the bulk, the atomic electric field would be suppressed by the superposition of the neighboring atomic electric fields. Conversely, the atomic electric field would not be suppressed when the atom is placed at a longer interatomic distance or a smaller coordination number. Figure 2a shows some topological defects such as five- or eight-membered rings and a part of a nanopore. Several C atoms at the eight-membered ring or the nanopore (red dots in Fig. 2a, c) are bonded with only two neighboring carbon atoms. Owing to the small coordination number, the net atomic electric field strength of these carbon atoms becomes stronger, and the electric field strength is enhanced normal to the C–C bond direction as indicated by the white arrows. Conversely, in the five-membered ring, the interatomic distances are shorter (strong covalency) and the observed atomic electric field is strongly suppressed, as seen in the faint dark contrast in Fig. 2b.

### Atomic electric fields at Stone–Wales defects and nanopores

**Fig. 3:**
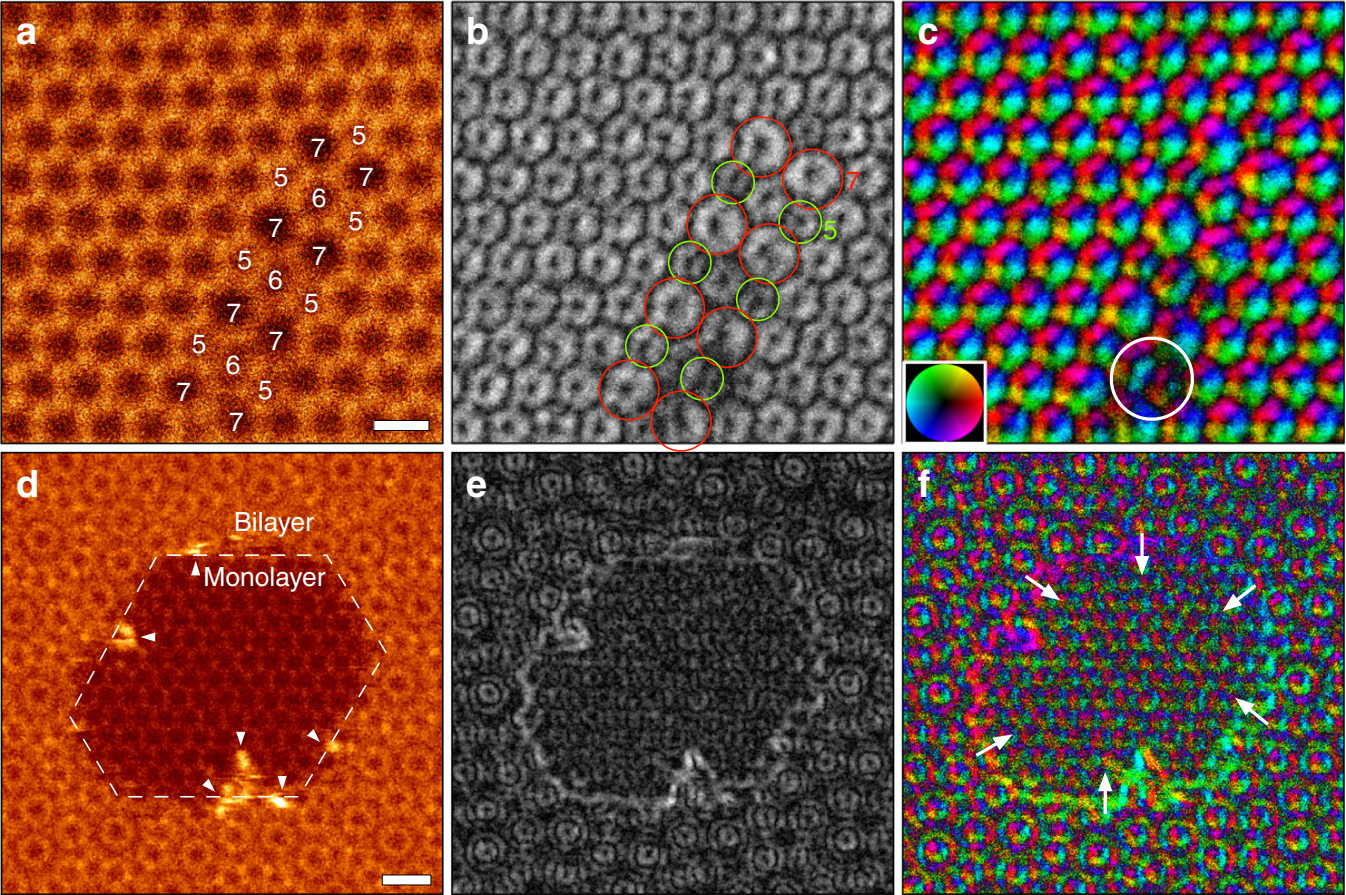
Atomic-resolution ADF and electric field maps at defects in graphene. **a**, **d** ADF-STEM images (512 × 512, 40 μs/pixel), **b**, **e** electric field strength maps, **c**, **f** electric field vector maps obtained from Stone–Wales defect and nanopore regions, respectively. The contrast ranges are (**b**) 0–25 V, (**e**) 0–45 V. The white arrows in (**f**) indicate the direction of the electric field. The scale bars in (**a**), (**d**) are 3 Å and 5 Å, respectively

To demonstrate the validity of our description for electric field images, we observed various topological defects in graphene. Figure 3a–c shows the simultaneously recorded ADF-STEM image, electric field strength, and vector maps obtained from Stone–Wales defects (5-7-5-7)^25^, where four sets of Stone–Wales defects are smoothly connected by the insertions of six-membered rings. The carbon atoms at the five-/seven-membered rings have shorter/longer bond lengths than that of the hexagonal ring, and the electric field strength certainly exhibits weaker/stronger contrast within their rings, respectively (green/red circles), in agreement with our description. The bond length between C atoms in a five-membered ring is slightly shorter than that of a seven-membered ring and we may need to consider the effect of contrast transfer function (CTF) of DPC–STEM imaging for the contrast change^26^. However, the estimated contrast reduction in a five-membered ring is only a few percent and therefore the effect of CTF is negligibly small. For further confirmation of the validity of our interpretation, we performed quantitative intensity analysis and image simulation with the DFT-derived structure model of Stone–Wales defect (see Supplementary Figures 2–4). These results well reproduced the experimental electric field strength. The larger electric field within the seven-membered ring could easily collect cations at the hollow site, and therefore the Stone–Wales defect can be considered a chemically active nano-field, especially for a catalytic reaction with metal ions. Here, we note that some atomic reconfigurations were observed at the region indicated by a white circle in Fig. 3c, which gives dark contrast in Fig. 3b, c.

During the observations, the high chemical reactivity of nanopores with cations was readily realized by the fast growth of nanopores. As we have noted, Stone–Wales defects or larger membered rings have a strong capability to capture single cations which then act as a catalyst to the etching and growth of nanopores^27^. Figure 3d–f shows a single frame of nanopores formed in bilayer graphene edge (with a twist angle of about 30°)^28^. The strong electric field was observed at the facet graphene edge in Fig. 3e and the electric field points toward the center of the nanopore, which can be clearly seen as the color gradation along the edge in Fig. 3f (white arrows). We note that the enhancement of the projected electric field at the graphene edge is roughly estimated to be ~20% (see Supplementary Figure 5). At the beginning, single Si atoms were trapped at the hollow site or vacancies and then the nanopore size gradually grew by the etching with the aid of electron beam irradiation. Over the period of probe scanning of this region, additional Si atoms were also trapped at the nanopore and the graphene edge became partly decorated by Si atoms, as shown by the bright contrast (white arrowheads) in ADF-STEM image of Fig. 3d. Since the electric field at the graphene edge (or dangling bond) is toward the center of the nanopore, the Si atoms could not climb out from the nanopore and so stayed over around the perimeter of graphene edge, promoting the growth of the nanopore. In addition to the presence of a dangling bond of C atoms, the attached Si atoms at the graphene edge enhance the local electric field as shown in Fig. 3e, which attracts the residual gas of oxygen or hydrogen and consequently the etching rate with Si atoms may be increased^29^. If we can completely decorate the graphene edge by cations^30^, the local electric field should be much enhanced, supplying an intriguing chemically active nano-field. Thus, direct electric field imaging of graphene defects should be a very powerful method to experimentally understand their chemical stabilities and activities from the atomic scale.

## Discussion

We have demonstrated the electric field mapping of atomistic defects in graphene, including single Si dopants in threefold and fourfold configurations, Stone–Wales defects, and nanopores by atomic-resolution DPC–STEM imaging. The electric field images can be considered as showing a force field formed by constituent atoms, which makes it possible to understand the local chemistry such as bonding orientation and the local chemical activity. Our findings show how electric field imaging can be used to investigate stable adatom sites and the growth of nanopores. Since the resultant signals in DPC–STEM imaging are mostly very small, we must carefully select the optimal experimental conditions such as accelerating voltage, beam current, and dwell time. The present method of multiple-frame averaging with a relatively low-dose condition could be helpful to suppress the electron beam damage. By properly choosing the experimental condition, the present electric field imaging is applicable to many other systems, and may also be helpful to identify the ion diffusion pathways in ionic conductive materials (the minimum electric field path) by experiments.

## Methods

### Atomic-resolution electron microscopy

We used a commercially available monolayer graphene (Graphenea, Spain), grown by chemical vapor deposition and then transferred onto a TEM grid. In this growth process, the sample was exposed in some Si sources, which provides Si dopants in graphene. To remove hydrocarbon contamination, the TEM grid was annealed at 400 °C for 3 h in a high vacuum TEM via in situ TEM heating holder (JEOL, Ltd), and then allowed to cool down to room temperature. Atomic-resolution ADF and DPC STEM images were acquired with a JEM 300CF installed in the University of Tokyo, equipped with a JEOL ETA corrector, cold field emission gun, and a second generation of SAAF detector (DPC STEM) operated at 80 kV. The used probe current was ~23 pA, as measured by a Faraday cup. The probe-forming aperture was 27 mrad in semi-angle (the expected probe size is 1.1 Å in full-width-half-maximum), the ADF detector spanned 42–170 mrad, and the bright-field disk edge was set in the middle of the third ring of the SAAF detector^12^. For the construction of the atomic electric field, we approximate the center of mass based on the signals in the 16 detector segments^17^.

### Image simulations

To obtain the stable defect structure models of Si–C_3_ and Si–C_4_ configurations, we performed DFT calculations using the VASP code^31^. The image simulations were performed using an absorptive model for thermal scattering, and imaging parameters consistent with the instrument configuration used: a 80-kV probe, a 27-mrad probe forming an aperture semi-angle, a chromatic aberration coefficient of 0.89 mm, and a Gaussian energy spread with a full-width-half-maximum of 0.45 eV. Effective source size was incorporated via convolution with a 2D pseudo-Voigt-like function, comprising a 20% weighting of a Gaussian with half-width-half-maximum of 0.39 Å and an 80% weighting of a Lorentzian with half-width-half-maximum of 0.47 Å as determined by fitting to the experimental ADF-STEM images.

## Electronic supplementary material

Supplementary Information

## Data Availability

The presented data are available from the corresponding author upon request.

## References

[1] Geim AK, Novoselov KS (2007). The rise of graphene. Nat. Mater..

[2] Dean CR (2013). Hofstadter’s butterfly and the fractal quantum hall effect in moire superlattices. Nature.

[3] Wang L (2017). Fundamental transport mechanisms, fabrication and potential applications of nanoporous atomically thin membranes. Nat. Nano..

[4] Kühne M (2017). Ultrafast lithium diffusion in bilayer graphene. Nat. Nano..

[5] Banhart F, Kotakoski J, Krasheninnikov AV (2011). Structural defects in graphene. ACS Nano.

[6] Krivanek OL (2010). Atom-by-atom structural and chemical analysis by annular dark-field electron microscopy. Nature.

[7] Suenaga K, Koshino M (2010). Atom-by-atom spectroscopy at graphene edge. Nature.

[8] Zhou W (2012). Direct determination of the chemical bonding of individual impurities in graphene. Phys. Rev. Lett..

[9] Ramasse QM (2013). Probing the bonding and electronic structure of single atom dopants in graphene with electron energy loss spectroscopy. Nano. Lett..

[10] Shibata N (2012). Differential phase-contrast microscopy at atomic resolution. Nat. Phys..

[11] Müller K (2014). Atomic electric fields revealed by a quantum mechanical approach to electron picodiffraction. Nat. Commun..

[12] Shibata N (2017). Electric field imaging of single atoms. Nat. Commun..

[13] Pauling L (1960). The Nature of the Chemical Bond.

[14] Shibata N (2010). New area detector for atomic-resolution scanning transmission electron microscopy. J. Electro Microsc..

[15] Sawada H, Shimura N, Hosokawa F, Shibata N, Ikuhara Y (2015). Resolving 45-pm-separated si–si atomic columns with an aberration-corrected stem. Microscopy.

[16] Pennycook SJ, Boatner LA (1988). Chemically sensitive structure-imaging with a scanning transmission electron microscope. Nature.

[17] Close R, Chen Z, Shibata N, Findlay SD (2015). Towards quantitative, atomic-resolution reconstruction of the electrostatic potential via differential phase contrast using electrons. Ultramicroscopy.

[18] Müller-Caspary K (2017). Measurement of atomic electric fields and charge densities from average momentum transfers using scanning transmission electron microscopy. Ultramicroscopy.

[19] Girit CO (2009). Graphene at the edge: stability and dynamics. Science.

[20] Ishikawa R, Lupini AR, Findlay SD, Pennycook SJ (2014). Quantitative annular dark field electron microscopy using single electron signals. Microsc. Microanal..

[21] Feynman RP (1939). Forces in molecules. Phys. Rev..

[22] Nakada K, Ishii A (2011). Migration of adatom adsorption on graphene using dft calculation. Solid State Commun..

[23] Kotakoski J, Mangler C, Meyer JC (2014). Imaging atomic-level random walk of a point defect in graphene. Nat. Commun..

[24] Susi T (2014). Silicon–carbon bond inversions driven by 60-kev electrons in graphene. Phys. Rev. Lett..

[25] Stone AJ, Wales DJ (1986). Theoretical studies of icosahedral C_60_ and some related species. Chem. Phys. Lett..

[26] Seki T (2017). Quantitative electric field mapping in thin specimens using a segmented detector: Revisiting the transfer function for differential phase contrast. Ultramicroscopy.

[27] Liu Z (2014). In situ observation of step-edge in-plane growth of graphene in a stem. Nat. Commun..

[28] Ishikawa R (2016). Interfacial atomic structure of twisted few-layer graphene. Sci. Rep..

[29] Wang X, Dai H (2010). Etching and narrowing of graphene from the edges. Nat. Chem..

[30] Lee J (2014). Stabilization of graphene nanopore. Proc. Natl. Acad. Sci..

[31] Kresse G, Furthmüller J (1996). Efficient iterative schemes for ab initio total-energy calculations using a plane-wave basis set. Phys. Rev. B.

