# Supplementary material for: Direct electric field imaging of graphene defects
Source: Nat Commun. 2018 Sep 24;9:3878. doi: 10.1038/s41467-018-06387-8 (PMC6155138; doi:10.1038/s41467-018-06387-8)
Supplement: Supplementary file 1 — Supplementary Information [file 41467_2018_6387_MOESM1_ESM.pdf]

Supplementary Information

# Direct electric field imaging of graphene defects


Ryo Ishikawa[1,*], Scott D. Findlay[2], Takehito Seki[1], Gabriel Sánchez-Santolino[1], Yuji Kohno[3], Yuichi Ikuhara[1,4], Naoya Shibata[1,4]

[1]*Institute of Engineering Innovation, University of Tokyo, Bunkyo, Tokyo, 113-8656, Japan*

[2]*School of Physics and Astronomy, Monash University, Victoria 3800, Australia*

[3]*Electron Optics Division, JEOL Ltd., Akishima, Tokyo, 196-855, Japan*

[4]*Nanostructures Research Laboratory, Japan Fine Ceramics Center, Nagoya, Aichi 456-8587, Japan*

[*]Correspondence and requests for materials should be addressed to R.I. (e-mail: ishikawa@sigma.t.u-tokyo.ac.jp)


## Supplementary Note 1. Projected electric field strength profile along the bonding direction.

To show the suppression of the atomic electric field between Si and C atoms, we plot the electric field strength along the bonding direction in Supplementary Figure 1c, d for the $SiC_3$ and $SiC_4$ configurations, respectively. The raw data of the electric field strength images are relatively noisy and so for the images given in Supplementary Fig. 1a, b we applied Gaussian convolution to enhance their interpretability. Along the X-X' direction, there are four intensity reductions in the profile of Supplementary Fig. 1c. Three intensity dips correspond to the H6-site, the T-site (or C atom position) and the Si atom position, respectively. The remaining dip, marked by the arrow, corresponds to the suppression of electric field between Si and C atoms. In the same manner, the electric field reduction related to the bonding between Si and C atoms can be seen the intensity dip in the profile (along the Y-Y' direction) of Supplementary Fig. 1d for the $SiC_4$ configuration.

## Supplementary Note 2. The effect of contrast transfer function for electric field at Stone-Wales defect

The bond lengths between C atoms in five-membered ring (1.38 Å) are slightly shorter than that of seven-membered ring (1.46 Å), and it is therefore required to consider the effect of contrast transfer function (CTF) for DPC-STEM imaging. Supplementary Figure 2 shows the CTF for DPC-STEM as a function of normalized spatial frequency with our segmented detector configuration, where the detailed theoretical background is given in Ref. 26. The contrast

transfer for five- or seven-membered ring corresponds to the red or blue lines in Supplementary Fig. 2, respectively, and the contrast reduction by optics is estimated to be about 3%, compared to that of seven-membered ring. While in experiment of Supplementary Fig. 3, the electric field strength in five-membered ring is about 30% smaller than that of five-membered ring. Therefore, we conclude that the observed suppression or enhancement of the electric field at the defects are not from the effect of CTF.

To confirm the validity of electric field suppression or enhancement at the five- or seven-membered rings, we further performed image simulation with the relaxed structure model by DFT calculation. The observed Stone-Wales defects in Figure 3a of the main paper is fairly complicated, but for simplicity, we here use a single Stone-Wales defect including two sets of five/seven-membered rings. Supplementary Figures 4a, b shows the relaxed DFT structure model of Stone-Wales defect and the simulated ADF-STEM image. The carbon atom positions in the ADF-STEM image are an exactly matched with the input DFT structure and no significant contrast enhancement or reduction is observed. Supplementary Fig. 4c, d show the simulated projected atomic electric field strength and vector maps with the DFT structure model. Compared with six-membered rings, the electric fields are distinctly suppressed or enhanced at the five- or seven-membered-rings, respectively.

**Supplementary Note 3. Enhancement of the projected Electric field at the graphene edge.**

To quantitatively evaluate the enhancement of the projected electric field strength at the graphene edge of the nanopore, we investigated the electric field strength across the graphene edge of the nanopore. Supplementary Figure 4c show the intensity profile along X-X' direction in Supplementary Fig. 4b of the projected electric field strength map, and the highest peak intensity corresponds to the location at the graphene edge of the nanopore. The average of the projected electric field strength of the monolayer graphene is estimated to be ~14 V. Since the carbon atoms are not aligned along z-direction at the twisted bilayer region, the projected electric field becomes slightly smaller than the that of simply doubled monolayer graphene. Although it strongly depends on the local atomic configuration, we roughly estimate a ~20% enhancement of the projected electric field strength at the graphene edge, where the coordination number is 2.

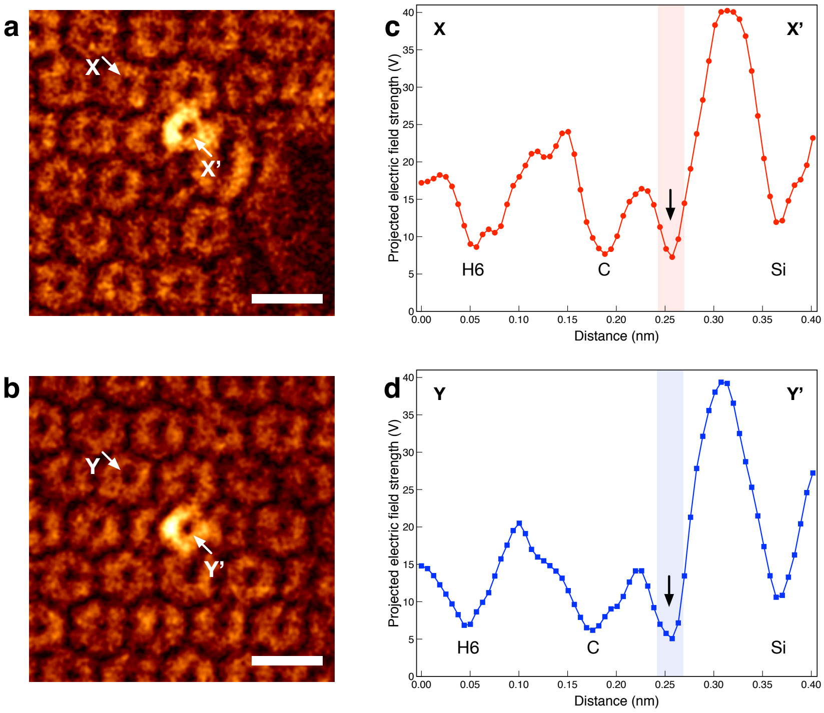


**Supplementary Figure 1 | Projected atomic electric field strength map for single Si dopants in graphene.** Electric field strength maps in the coordination of (a) $SiC_3$, (b) $SiC_4$. The intensity profiles (c) along the X-X' direction and (d) along the Y-Y' direction. The scale bars in (a), (b) are 3 Å, respectively.

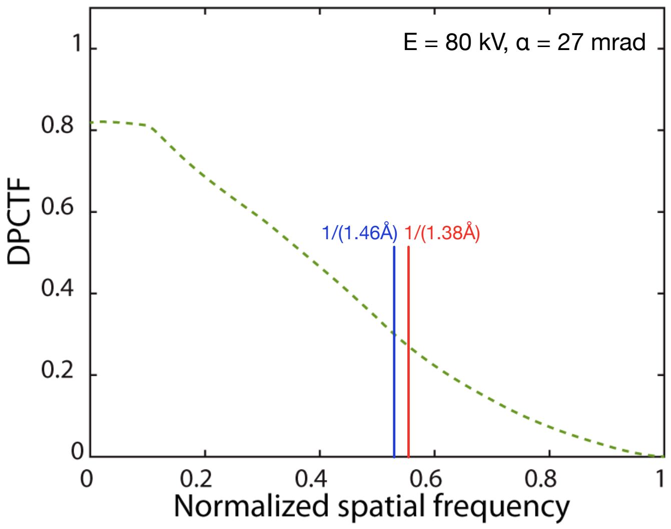


**Supplementary Figure 2 | CTF for DPC-STEM imaging.** CTF for DPC-STEM as a function of normalized spatial frequency for the present experimental condition (80 kV and 27 mrad). The red or blue lines correspond to the frequency for the 5 or 7-membered ring bond length, respectively.

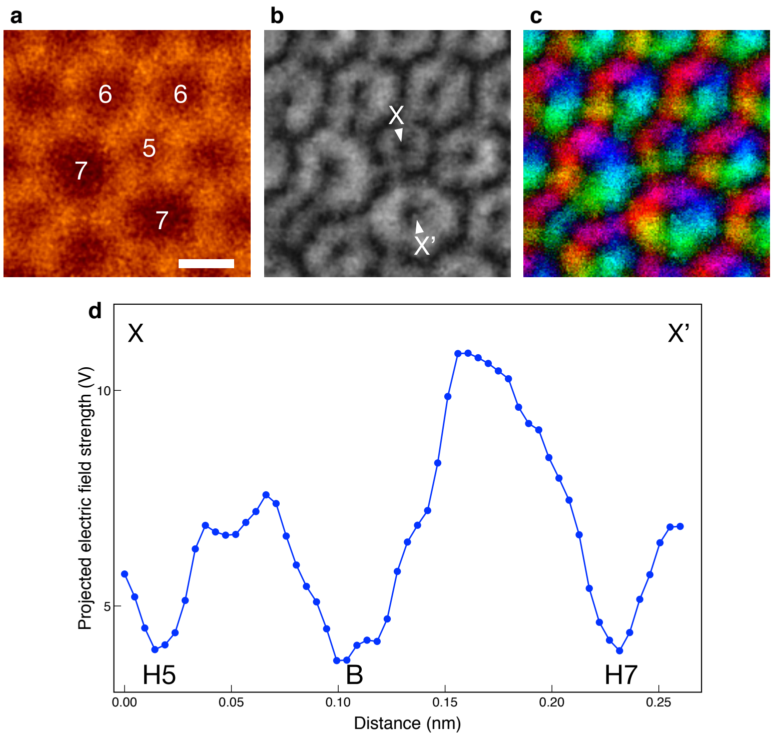


**Supplementary Figure 3 | Electric field strength at Stone-Wales defects.** (a) ADF-STEM, (b) electric field strength map, (c) electric field vector map obtained from Stone-Wales defect (magnified image of Fig. 3). (d) Electric field strength profile along the X-X' direction in (b), where the intensity dips correspond to H5- (hollow 5), B- and H7-sites, respectively. The scale bar in (a) is 2 Å.

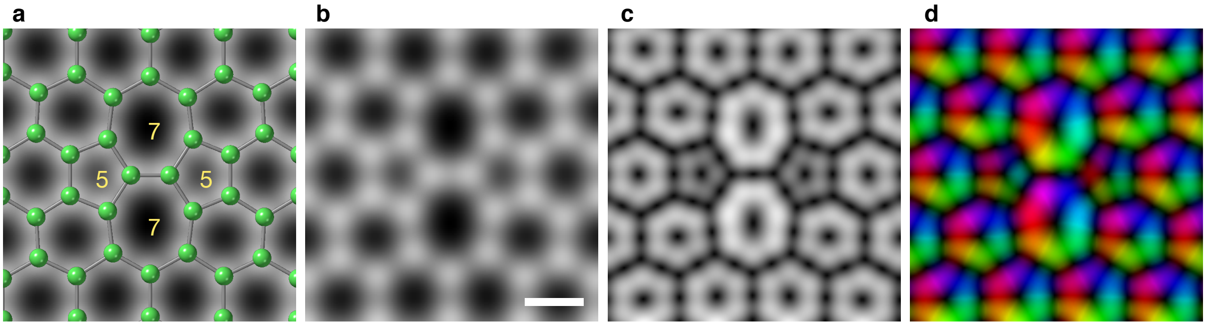


**Supplementary Figure 4 | Simulated projected atomic electric field maps at Stone-Wales defects in graphene.** (a) relaxed DFT structure model of Stone-Wales defect, (b) ADF-STEM image, (c) electric field strength map, (d) electric field vector map. The scale bar in (b) is 2 Å.

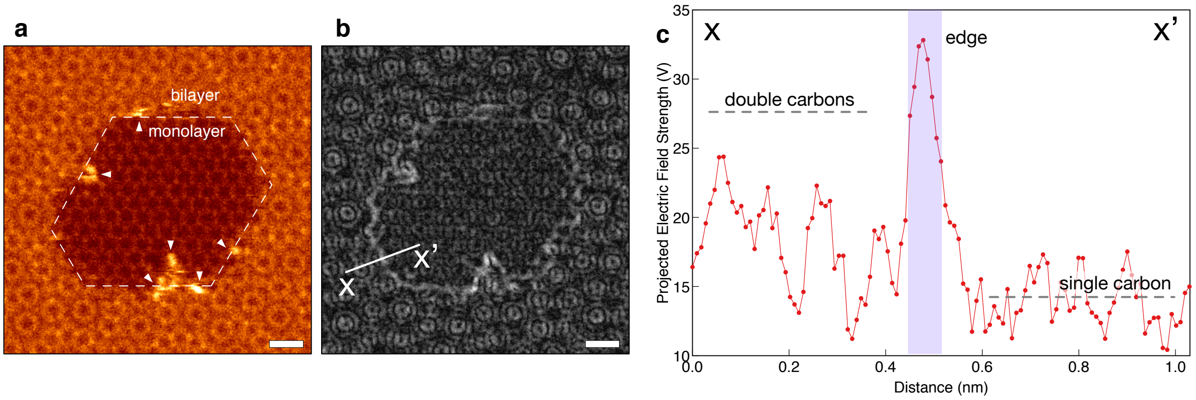


**Supplementary Figure 5 | Electric field strength at the bilayer graphene edge.** (a) ADF-STEM, (b) projected electric field strength, (c) intensity profile along the X-X' direction given in (b). The scale bars in (a), (b) are 5 Å, respectively.
